# Wireless Capacitive Liquid-Level Detection Sensor Based on Zero-Power RFID-Sensing Architecture

**DOI:** 10.3390/s23010209

**Published:** 2022-12-25

**Authors:** Shaheen Ahmad, Ramin Khosravi, Ashwin K. Iyer, Rashid Mirzavand

**Affiliations:** 1 Mechanical Engineering Department, University of Alberta, Edmonton, AB T6G 2R3, Canada; 2 Electrical and Computer Engineering Department, University of Alberta, Edmonton, AB T6G 2R3, Canada

**Keywords:** capacitive sensor, radio-frequency identification (RFID), interdigitated electrode capacitor (IDC), ultrahigh frequency (UHF), sensitivity, zero-power sensor, grounded coplanar waveguide (GCPW), battery-less, wireless sensor

## Abstract

In this paper, a new method for the wireless detection of liquid level is proposed by integrating a capacitive IDC-sensing element with a passive three-port RFID-sensing architecture. The sensing element transduces changes in the liquid level to corresponding fringe-capacitance variations, which alters the phase of the RFID backscattered signal. Variation in capacitance also changes the resonance magnitude of the sensing element, which is associated with a high phase transition. This change in the reactive phase is used as a sensing parameter by the RFID architecture for liquid-level detection. Practical measurements were conducted in a real-world scenario by placing the sensor at a distance of approximately 2 m (with a maximum range of about 7 m) from the RFID reader. The results show that the sensor node offers a high sensitivity of 2.15°/mm to the liquid-level variation. Additionally, the sensor can be used within or outside the container for the accurate measurement of conductive- or non-conductive-type liquids due to the use of polyethylene coating on the sensitive element. The proposed sensor increases the reliability of the current level sensors by eliminating the internal power source as well as complex signal-processing circuits, and it offers real-time response, linearity, high sensitivity, and excellent repeatability, which are suitable for widespread deployment of sensor node applications.

## 1. Introduction

Wireless sensing systems are becoming widespread due to the growth in the Internet of Things (IoT) industry and the development of new mobile communication technologies. Among different sensing applications, liquid-level measurement is one of the most widely studied techniques due to its practical use in many real-life applications, including smart homes, healthcare, petroleum, food, and agriculture. The increasing demand for the massive deployment of these sensors will stretch valuable energy and cost resources. Thus, researchers are focusing on developing new power-efficient sensing methods with reduced design complexity that can fulfill the requirements of the future sensing systems [1]. In this regard, passive radio-frequency identification (RFID)-based sensors are a viable solution due to their power efficiency, reliability, and design simplicity.

The use of conventional sensor nodes requiring an internal power source and complex signal-processing circuits are becoming costly and impractical for widespread deployment. Battery-less UHF RFID sensors, on the other hand, provide real-time continuous sensing measurement with high reliability and without the use of any internal power source or a complex signal-processing circuit, by utilizing the phase variation of the backscattered signal as a sensing parameter. These passive RFID sensing nodes use energy harvesting from the signal sent by the reading device that reduces cost and increases the reliability of the wireless sensor systems. In addition to tracking and identification, RFID technologies are receiving increasing popularity as a wireless sensing system in many industrial applications [2,3,4]. However, the conventional RFID tag design has to be modified for most of the modern sensing applications [5,6,7]. Therefore, a new robust sensing method needs to be established to use the current RFID systems for a reliable passive wireless measurement of liquid level.

The detection of liquid level is divided broadly into few categories such as classification based on the type of measurement, i.e., continuous level measurement [8] for process monitoring or point level measurement [9] for marking a single liquid-level height to activate an alarm. These sensors are further classified based on their contact [10] and non-contact [11] nature with the sensing medium. The accuracy of contact-type sensors is usually smaller due to their dependence on liquid properties that affect the sensor performance, mostly in the case of conducting type liquids; therefore, non-contact level measurement is widely explored in the literature. Although most of the work is focused on the sensitivity and detection range improvement, much less emphasis has been given to the development of low-power reliable sensing nodes that can be densely deployed for applications such as wireless IOT. Most of the liquid-detection sensors need a wired connection or a built-in battery to operate, which increases the design complexity and cost and requires regular maintenance, making them unsuitable for many modern sensing applications [12].

Several liquid-level detection sensors have been presented in the past based on multiple design techniques. Coaxial cylindrical probe sensors are widely known level sensors that have reasonable accuracy and linearity, but they are bulky and require a high amount of power to operate [13]. Optical [14] and ultrasound [15] sensing techniques provide high-resolution liquid-level measurement; however, the optical technique has high manufacturing and maintenance costs as well as health and safety issues, while the ultrasound measurement is mostly erroneous due to its sensitivity to environmental conditions. Microwave resonators [16], acoustic wave resonators [17], interdigitated electrodes [18], and comb electrodes [19] are other popular techniques for liquid-level measurement; however, they utilize complex digital circuits for signal processing that require additional power to operate, resulting in lower power efficiency, increased cost, and increased design complexity.

Recently, some efforts were made to use a chipless RFID technology as a liquid-level detection system with non-linear behavior and a short detection range below 50 cm [20]. Several other passive RFID-based sensors have been developed that can detect liquid within the container but lack the capability of providing continuous information of the liquid-level variation in real time [18,21,22]. Sensing systems based on semi-passive RFID technology have been the focus of many researchers [23,24,25], due to their higher read-range as compared to the passive RFID systems. Despite the fact they use signals from the reader to energize the chip, semi-passive RFID technology also requires an internal power source to drive the electronic circuits, making them unreliable, bulky, and costly. Some other types of battery-less RFID systems available on the market incorporate digital circuitry that can add the sensed information digitally to the backscattered signal with better accuracy and with the capability to integrate off-the-shelf sensors; however, the power required by the digital circuits significantly reduces the sensing read-range [26,27]. In [28], a passive RFID-chip-based design is presented with a read range of up to 8 m; however, it can only be used as a flood sensor without providing any information about the liquid level height.

**Figure 1:**
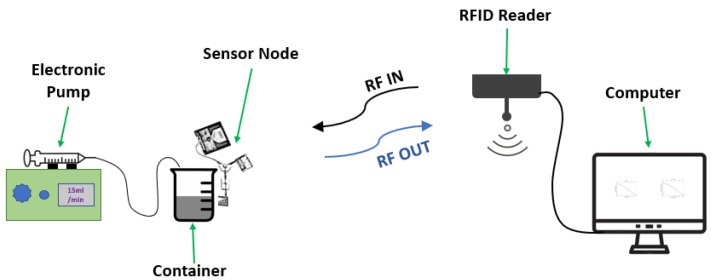
Demonstration of the proposed wireless liquid-level measurement system.

In this paper, we propose a novel method for the passive wireless detection of liquid level by combining the advantages of the zero-power UHF RFID systems with a highly sensitive capacitive-sensing element to address the aforementioned challenges. The design comprises a three-port UHF RFID-sensing architecture and a capacitive-sensing element. The working principle is based on a variation in fringe capacitance of the sensing element due to the changes in the liquid level; this introduces an extra phase delay to the backscattered signal, which is received by the RFID reader. The variation in the liquid level causes a high phase transition due to the resonance frequency (reactive capacitance) shift of the capacitive-sensing element at a passband frequency of 927 MHz, which is extracted at the reader by subtracting the phase variation from the reference phase of the stop band frequencies. The performance of the sensing system is evaluated in a real-life scenario as shown in Figure 1. Reactive phase change measurement is a simple, inexpensive sensing technique offering some additional advantages such as short response time, stability, and low power consumption, which are suitable for low-power-cost, effective wireless sensing systems. The proposed liquid-level detection system increases the reliability of the current level detection sensors by integrating a highly linear reactive element with a passive sensing node to reduce periodic maintenance, cost, and design complexity and to increase sensitivity and measurement linearity. The experimental results demonstrate that very good linearity, sensitivity, and repeatability can be achieved by the proposed capacitive zero-power RFID sensing system.

The goals of this research are three-fold:

- Propose a new sensing method for the wireless detection of liquid level with high sensitivity;
- Design and integrate a highly sensitive reactive sensing element with a passive RFID architecture to form a wireless level measurement system;
- Demonstrate the measurement system performance in a real-world scenario.

The rest of the paper is organized as follows: Section 2 explains the design and working principle of the zero-power wireless sensor architecture and the capacitive-sensing element. Section 3 includes details about the experimental setup along with the discussion and validation of the measured result. Finally, the conclusion is provided in Section 4.

## 2. Theory

### 2.1. Sensor Architecture

**Figure 2:**
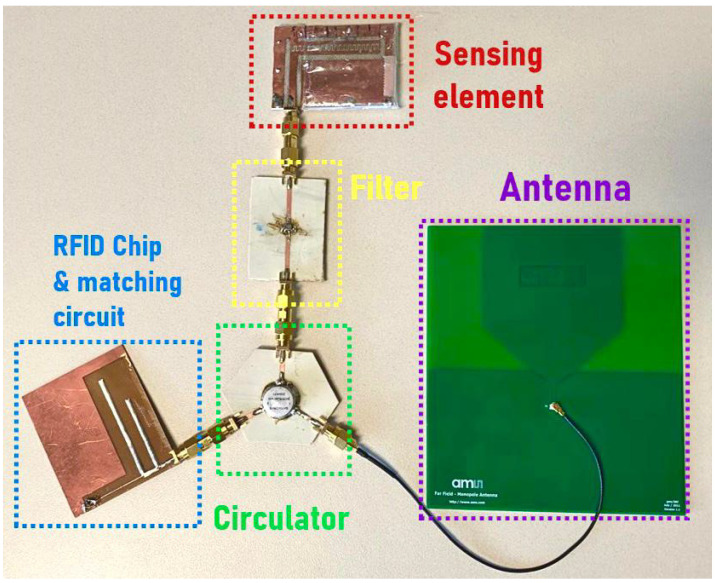
Three-port RFID sensing node.

The proposed sensing method integrates a capacitive-sensing element with a passive three-port UHF RFID architecture to develop a wireless liquid-level detection system. The three-port architecture has been previously developed and tested for other sensing applications [28,29]. The sensing system comprises a circulator, a filter, a UHF RFID chip, and an antenna, as shown in Figure 2. Due to the non-reciprocal nature of the circulator, the signal received from the reader by the antenna is sent to the RFID chip for excitation. Upon energizing, the chip reflects a backscattered signal that is then diverted to the reactive sensing element through a bandpass filter. The sensing element adds a phase delay to the signal in the passband (927 MHz) based on the information from the liquid-level height, which is received by the reader. The phase of the reflected frequencies in the filter stopband is used as a reference to calculate the phase difference caused by the change in the liquid level. These backscattered signals, when received by the reader, are used to generate IQ signals with the help of a built-in noncoherent IQ demodulator. By programing the reader, the phase delay caused by the sensing element due to the variation in the liquid level can be determined by subtracting the phase delay of the pass-band frequencies from the reference phase of the reflected signals. The reference phase is helpful to remove any phase ambiguity due to the distance of the wireless node from the RFID reader as well as the phase delay added by various components in the architecture. The reference phase in the filter stop-band also reduces the measurement uncertainties caused due to the reflections from obstacles and simplifies the measurement method by eliminating the need for distance-specific calibration.

### 2.2. Sensing Element Design and Specifications

Interdigitated electrode sensors have been studied in the past for sensing applications, including their use in liquid-level detection [30,31,32]. The corresponding change in the capacitance of these structures can be used to determine the changes in the material under observation. The capacitance variation, however, needs to be converted to a measurable parameter such as current, voltage, etc., using an additional signal-processing circuit that requires an extra amount of power to operate. Thus, an internal power source is needed to drive these circuits that increase the cost and complexity of the sensor nodes. Since RFID-based sensing can utilize phase variations as a sensing parameter without the use of any additional circuitry; therefore, our focus is to optimize phase variation caused due to the corresponding changes in the capacitance of the interdigitated electrode structure. This capacitance variation alters the phase of the backscattered RFID signal at a fixed pass-band frequency, which is sent back to the reader with additional phase delay based on the liquid-level variation. Usually there is a high phase transition associated with the resonance frequency shift. The designed sensing element is optimized to have a resonance at 927 MHz frequency, the magnitude of which changes due to the variation in the liquid level. We exploit the high phase transition caused by the variation in the magnitude of the sensing element’s resonance as a sensing parameter to detect changes in the liquid level by integrating the IDC structure with an RFID-based sensing architecture.

**Figure 3:**
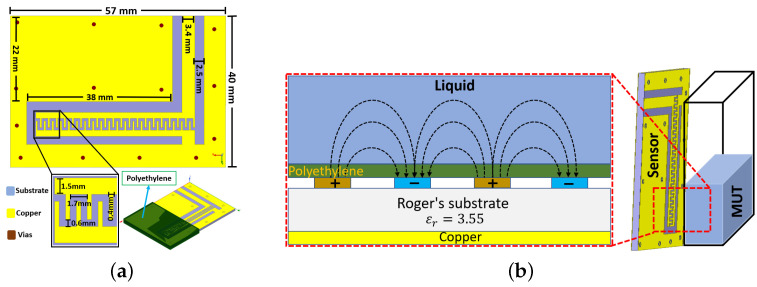
(**a**) Diagram of the proposed sensing element. (**b**) Schematic representation of the sensing element.

The sensing element is designed using grounded coplanar waveguide (GCPW) feeding, as shown in Figure 3a. The structure is designed and optimized using Ansys full-wave FEM software on a Rogers RO4003 substrate ($\epsilon_{r}=3.55$) with an overall dimension of 57 mm × 40 mm × 1.6 mm. Electrode fingers with positive potential are fed by the feedline, while the negative electrodes terminal is directly shorted to the ground. The active part of the sensor is coated with an approximately 600 μm-thick polyethylene layer to make it suitable for use in both conductive- and non-conductive-type liquids, as well as to use it as a contact or a non-contact sensor. The optimized design is fabricated using an LPKF U3 laser-based and mechanical-milling-based ProtoMat S62.

The analytical equation for calculating the IDC capacitance is well known; it depends on several parameters such as the dimensions of the IDC, the gap between the electrodes, the substrate material, and the properties of the material under test (MUT). The MUT in this case is either air, liquid, or a combination of both, and it depends on the liquid height. For a minimum liquid level, most of the electric fields pass through the air, while for maximum level the fields pass through the liquid sample, changing the sensor capacitance. Figure 3b shows a simplified schematic of the proposed sensing element with the representation of the fringing fields passing through the test medium. The mathematical expression for the IDC capacitance has been studied and reported by several authors in the literature [33,34]. The capacitance formula for the IDC capacitor is given by [34],

(1) $$C=L\left(N-1\right)\left(\frac{\epsilon_{0}\epsilon_{r,t}}{2}\frac{K\sqrt{1-k^{2}}}{K(k)}+2\epsilon_{0}\epsilon_{r,m}\frac{h}{d}\right),$$

where *L* is the length of the electrodes, *N* is the number of electrodes; $\epsilon_{0}$ is the permittivity of vacuum $8.854\times 10^{-12}$ F/m; $\epsilon_{r,t}$ is the relative permittivity of the test material; *h* and *w* are the thickness and width of the electrodes, respectively; *d* is the spacing between the electrodes; and *K(k)* is the first-order elliptical integral to calculate the impact of fringing fields, where *k* can be represented by,

(2) $$k=\cos\left(\frac{\pi }{2}\frac{w}{d+w}\right).$$

## 3. Results and Discussion

The feasibility of the proposed system was tested by implementing the model in Keysight’s advanced design system (ADS). The sensing element was designed, simulated, and optimized using Ansys full-wave FEM simulations to have high linearity, maximum sensitivity, and fast response to change in the liquid level. In the simulation model, phase variation with respect to liquid level was plotted at a fixed UHF frequency of 927 MHz.

**Figure 4:**
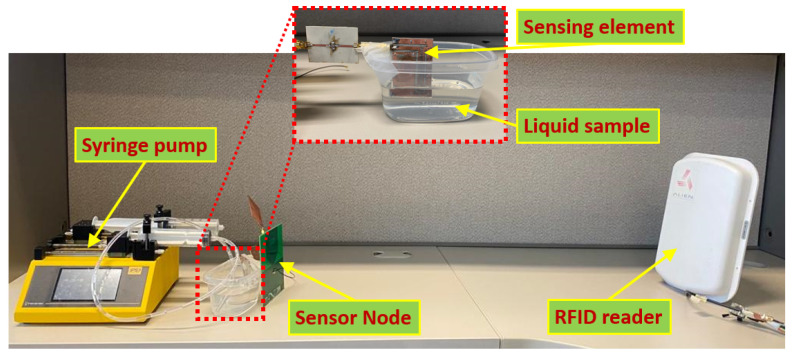
Experimental setup for wireless liquid level measurement.

The sensing element was then integrated into the wireless sensing architecture described in Section 2.1 for real-time measurements of liquid level in a real-life scenario. The experimental setup in Figure 4 shows a sensor node placed at a distance of about 2 m (measurements up to approximately 7 m is possible) from the RFID reader assuming that there are no obstacles between the reader and the sensor node that can affect the line of sight communication. The sensing element was fixed inside a transparent liquid container and marked with a millimetre scale to detect the liquid-level height. The behavior of the sensing element was assumed to be stable as there was no change in the properties of the substrate and copper traces during the experiments. The liquid level inside the container was varied in a controlled environment using an electronic syringe pump (IPS-14RS) and pipes to add/remove liquid to/from the container. Tape water was used for all of the experiments at a room temperature of 22 °C. The change in the phase value observed by the sensor is sent wirelessly to the RFID reader through an antenna connected to the port 1 of the circulator. The AS3993 Fermi RFID reader used for this experiment generates IQ signals from the received backscattered signal utilizing a mixer, which is used for determining the phase variation due to changes in the liquid level. The reader is programmed in a way to plot the phase variation due to changes in the liquid level by subtracting the phase delay caused by the sensing element from the reference phase.

**Figure 5:**
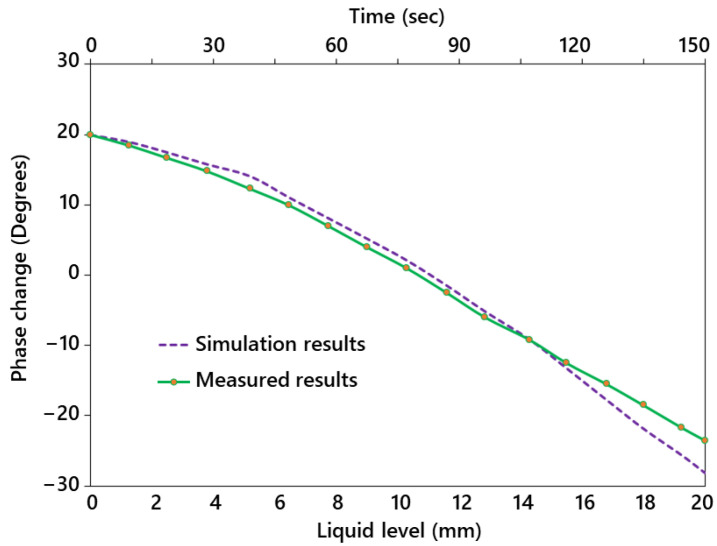
Sensor phase variation of the backscattered signal (measured) with respect to the liquid level, and a comparison of the simulated and measured phase variation.

Figure 5 shows the phase variation of the recorded backscattered signal at the reader as the liquid level changes. The initial sensor phase value was 20° at the 0 mm liquid level, which is linearly downshifted to −23° for a liquid height of 20 mm. The sensitivity of the sensor was found to be 2.15°/mm, which makes the sensor highly efficient for a wide range of applications. The sensor’s sensitivity to liquid variation depends on the polyethylene coating thickness over the sensitive part. Sensitivity increases with the decrease in the thickness of the polyethylene layer due to the increased number of fringing electric fields passing through the test medium. The tap water used for these experiments is considered to have constant properties, and the temperature is fixed during the tests. A comparison between the simulated and measured phase variation with respect to the liquid level demonstrates good agreement between both the results. The phase change in the simulations, however, is slightly higher than the measured results because of the additional parasitic capacitance during experimental measurements that reduces the overall phase variation of the sensor.

**Figure 6:**
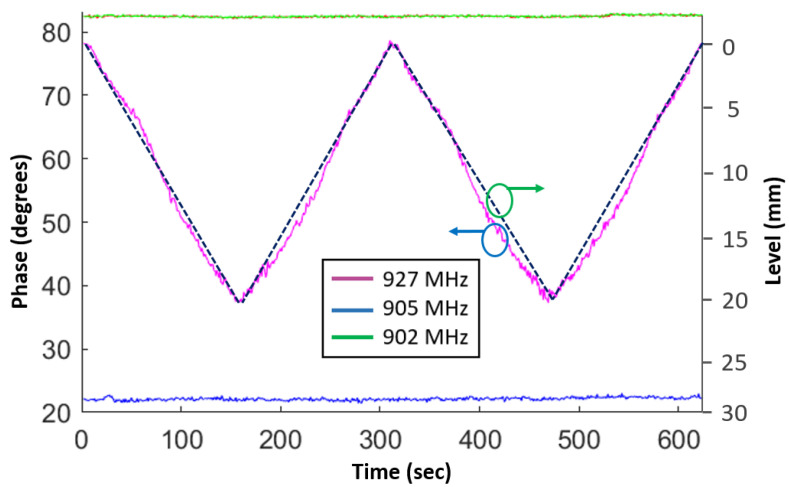
Repeatability test results of the proposed sensor.

A repeatability measurement of liquid level sensor is important to ensure proper operation over an extended period of time. A repeatability test was performed by increasing/decreasing the liquid level multiple time and plotting the phase variation with respect to time as well as liquid height, as shown in Figure 6. The sensor phase change due to liquid level was recorded at a fixed passband frequency of 927 MHz (pink). The phase values at two stopband frequencies of 902 MHz (green) and 905 MHz (blue) are also included, indicating a constant phase value according to the architecture-design principle, as explained in Section 2.1. The sharp edges of the triangular style graph at the maximum and minimum level indicate that the direction of phase changes abruptly when the liquid direction is reversed. Thus, the sensor offers good repeatability as well as a fast response to change in the liquid level.

**Figure 7:**
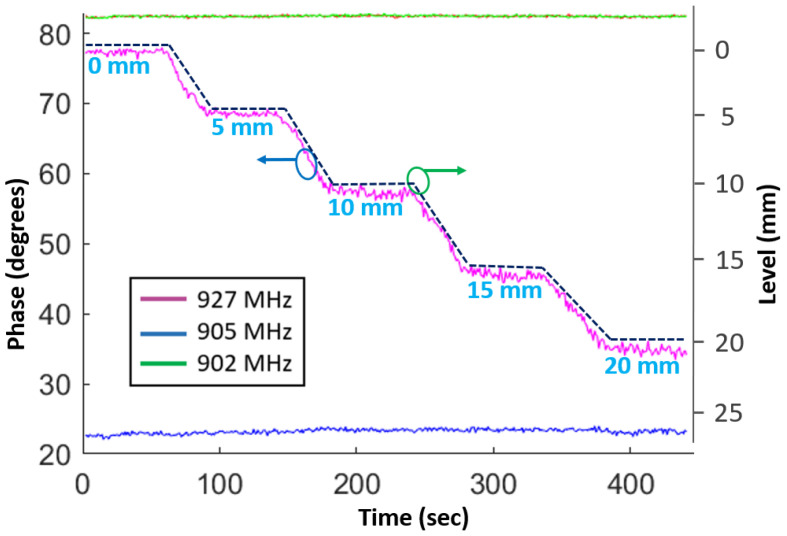
Sensor phase variation with respect to change in the liquid level in steps.

Furthermore, some additional tests were performed to ensure that the sensor can hold a constant phase value for different liquid heights. Figure 7 shows the phase variation of a backscattered signal by increasing and holding the liquid level at different heights. During each hold, the liquid level was kept constant for roughly 60 s and then increased in steps with a step size of 5 mm. From the figure, it can be seen that the sensor phase is constant when there is no change in the liquid level and decreases linearly with an increase in the liquid height. These experiments confirm that the proposed sensor can detect any height within the measurement range. Therefore, it can be used as a point-level detector as well as a continuous-level measurement wireless sensor.

A comparison of the proposed wireless sensing method with other liquid-level detection sensors is given in Table 1. The sensor can detect liquid level wirelessly from a remote location with high linearity. By using the phase of the RFID signal as a sensing parameter, the complex signal-conversion circuits at the sensor node are avoided to reduce the cost and complexity of the proposed level sensor. The sensitive part of the sensor is coated with a polyethylene layer, making it capable of being used as a contact or a non-contact sensor in conductive- or non-conductive-type liquids. During contact sensing, the liquid is in contact with the polyethylene coating only when there is no direct contact with the electrodes of the IDC structure, which reduces the dependence of the sensor performance on the liquid properties. The sensor also has the capability to be used as a non-contact sensor (if needed) placed outside of the liquid container, using the effect on the fringing electric fields due to changes in the liquid level as a sensing parameter. Additionally, the polyethylene coating removes the hysteresis effect due to its smooth surface, which does not allow any liquid to accumulate on the surface of the sensing element, especially when the liquid level is dropped, thus providing excellent repeatability. Although the sensor is designed to detect changes in the liquid level for up to 20 mm, it can fulfil the current and future requirements of this class of sensing applications in a densely populated wireless environment. The detection range can be easily extended from 20 mm to several centimeters by cascading the proposed sensing element design.

## 4. Conclusions

In this article, a capacitive IDC-sensing element is designed and integrated with three-port sensing architecture to devise a new method for wireless liquid-level measurement. The reactive capacitance of the sensing part changes with the liquid-level variation. A high phase transition associated with the change in the reactance is used as a detection parameter by the RFID sensor for liquid-level measurement. The active part of the sensor is coated with an approximately 600 μm-thick polyethylene layer, making it suitable for use in both conductive- and non-conductive-type liquids, and the sensing element of the sensor can be placed within or outside the container. Experimental results demonstrate a linear phase change response with a high sensitivity of 2.15°/mm. All of the experiments were performed at room temperature. The proposed sensor offers reliability, a real-time response, linearity, high sensitivity, and excellent repeatability, making it capable of use in a wide range of applications, especially IOT-based sensing environment.

## Figures and Tables

**Table 1:** Comparison of the proposed wireless sensor with other liquid-level detection sensors.

Refs.	Sensing Element	Complexity	Self-Powered	Wireless	Measurement Type	Sensitivity
[35]	Impedance phase	Moderate	No	No	Contact	0.21°/mm
[16]	Resonance	Low	No	No	Non-contact	-
[36]	Capacitive	Moderate	No	No	Contact	40.26 fF/mm
[37]	Magnetic coupling	Moderate	Yes	Yes	Contact	-
[38]	Resistive	Moderate	No	Yes	Non-contact	8.75 Ω/mm
[39]	Capacitive	Low	Yes	Yes	Contact/Non-contact	-
[40]	Coaxial cylindrical	Low	No	No	Contact	1.05 pF/mm
This work	Reactive phase	Low	Yes	Yes	Contact/Non-contact	2.15°/mm

## Data Availability

Data is contained within the article.
